# The impact of immediate breast reconstruction on the time to delivery of adjuvant therapy: the iBRA-2 study

**DOI:** 10.1038/s41416-019-0438-1

**Published:** 2019-03-29

**Authors:** Rachel L O’Connell, Tim Rattay, Rajiv V Dave, Adam Trickey, Joanna Skillman, Nicola L. P. Barnes, Matthew Gardiner, Adrian Harnett, Shelley Potter, Chris Holcombe, on behalf of the iBRA-2 Steering Group, the Breast Reconstruction Research Collaborative, Nicola L. P. Barnes, Jane Blazeby, Elizabeth Conroy, Rajiv V Dave, Matthew Gardiner, Adrian Harnett, Chris Holcombe, Ciara O’Brien, Rachel O’Connell, Shelley Potter, Tim Rattay, Joanna Skillman, Paula Williamson, Alain Curnier, Amir Tadros, Ivan Depasquale, Yazan A Masannat, Elizabeth Smyth, Mairi Fuller, Roger Bourne, Steven Heys, Ishrak Hamo, Fatima Aloraifi, Laura Fopp, Radhika Bali, Sarah Bache, Sarah L Benyon, Michael S Irwin, Amit Agrawal, Charles M Malata, Claire Murphy, Adam Misky, Dennis Wayne Chicken, Nassreen Abdullah, Arnold D K Hill, Carolyn Cullinane, Gareth Irwin, Stuart A McIntosh, Sigi Refsum, Samantha Sloan, Peter Mallon, Chiara Sirianni, Ilyas Khattak, Chiara Sirianni, Geerthan Nagachandra, Pasupathy Kiruparan, Debasish Debanth, Simon Davey, Terry-Ann Curran, Matilda Svenning, Sasirekha Govindarajulu, Zenon Rayter, Rachel Ainsworth, Simon Cawthorn, Ajay Sahu, Sherif Wilson, Elena Prousskaia, Antonello Accurso, Nicola Rocco, Rosa Di Micco, Antonello Accurso, Gennaro Limite, Raffaele Ceccarino, Raffaele Liccardo, Guido Coco, Metin Nizamoglu, Mary Morgan, Venkat Ramakrishnan, Giuseppe Catanuto, Alex Wilkins, Penelope McManus, Peter Kneeshaw, Kartikae Grover, Tapan Mahapatra, Brendan Wooler, Bilal Elahi, Naila Ihsan, Alexandra Bucknor, Dimitris Reissis, Judith Hunter, Simon Wood, Navid Jallali, Francis P Henry, Liaquat S Verjee, Jason Lee, Shazia M Khan, Iman Azmy, Julia Massey, Ciaran Hollywood, Michael Oluwajana, Sonia Bathla, Joanna Seward, Claudia Harding-MacKean, Risha Lane, Kothandaraman Murali, Bashishta Biswas, Pawel Trapszo, Seema Seetharam, Katy Kennedy, Louise Alder, Tomasz Graja, Khalid Amin, Jalal Kokan, Chandeena Roshanlall, Emma Gill, Dhananjay Kulkarni, J. M. Dixon, Oliver Young, Talha Saleem, M Biddle, Marie Kearns, Eva Weiler-Mithoff, Ben Chew, Andy Malyon, John Scott, David McGill, Iain Mackay, Salena Bains, Sara Barrows, Tim Rattay, Simon Pilgrim, Sheila Shokuhi, Kelly Lambert, Frances Kenny, Kalliope Valassiadou, Monika Kaushik, Jaroslaw Krupa, Dimitris Dragoumis, Quratul Ain, Pavlos Lampropoulos, Sarah Moss, Haitham Khalil, Anwar Haq, Balapathiran Balasubramanian, Petros Charalampoudis, Hisham Hamed, Ashutosh Kothari, Tibor Kovacs, Michael Douek, Iftikhar Mehmood, Biswajit Ray, Matthew Adelekan, Laura Humphreys, Salim Tayeh, Christina Choy, Laila Parvanta, Silvia Michieletto, Tania Saibene, James O’Brien, Sue Down, Sarah Downey, Jerome Pereira, A S Sami, Anzors Gvaramadze, Jibril A Jibril, Dinesh Thekkinkattil, S Udayasankar, Saira Khawaja, Yousef Shariaha, Simon Holt, Ruth James, Hirah Rizki, Katharine Kirkpatrick, Duraisamy Ravichandran, Deepak Shrestha, Ellora Barua, Deepika Akolekar, Ahmed Hamad, Eleftheria Kleidi, Susan Hignett, Vanessa Pope, Salma Naseem, Jennifer Isherwood, Rachel Soulsby, Amanda Taylor, Kian Chin, Dai Nguyen, Francesca Guest, Amanda Thorne, Valentina Lefemine, Chris Kirchhoff, Declan C Murphy, Michelle Lo, Ruth Harcourt, Simon J Pain, Maged I Hussien, Katalin Zechmeister, E. M. Sassoon, Andrea Figus, Richard M Haywood, Rozina Ali, Susanna Alexander, Adrian Harnett, Konstantinos Geropantas, Daniel Epurescu, Rebecca Lewis, Oladapo Fafemi, Jasdeep Gahir, Tasha Gandamihardja, Jennett Kelsall, Nazli Muhibullah, Charlene Otieno, Fayyaz Mazari, Marta Dauria, Lisa Whisker, Douglas Macmillan, Eleanor Gutteridge, Tuabin Rasheed, Hazem Khout, Kristjan Asgeirsson, Stephen McCulley, Maria Donatella Mariniello, Manuela Roncella, Matteo Ghilli, Livio Colizzi, Elena Rossetti, Lo Russo Marzia, Loredana Fustaino, Alessandro Quattrini Li, Kate L Harvey, Rebecca Windle, Dionysios Dennis Remoundos, Pankaj Roy, Gael MacLean, Asha Adwani, Elena Popa, Steven Goh, Geeta Shetty, Sarah Clark, Lorenzo Bernaudo, Avi Agrawal, Lucy Mansfield, Sally Tebbal, Ashraf Patel, Veronica Grassi, Ojas Pujji, Kathryn Hamnett, Naren Basu, Emily Granger, Michael Durbar, Panagiotis Pikoulas, Clare Garnsey, Philip Walker, Angela J Vollermere, Ioannis Michalakis, Robin Jones, Mina Youssef, Charlotte Ives, Mohammad Masood, Julie Dunn, Sisse Olsen, Douglas Ferguson, Rachel Tillett, Anna Allan, Alex Woollard, Rebecca Canny, Alexander Woollard, Afshin Mosahebi, Stephen Hamilton, Shadi Ghali, Daniel Marsh, Jagdeep Chana, Nilesh Sojitra, Ibby Younis, Dick Rainsbury, Natalie Chand, Vasileios Kalles, Anne Stebbing, Kevin Harris, Siobhan Laws, Chris Holcombe, Anne Tansley, Geraldine Mitchell, Emma de Sousa, Julia Henderson, Mysore Chandrashekar, Bernadette Pereira, Chloe Constantinou, Dalia Elfadl, Foivos Irakleidis, Izaro Hernan, Miriam Byrne, Natalie To, Rachel O’Connell, Jennifer Rusby, Peter Barry, Katerine Krupa, William Allum, Fiona MacNeill, Nicola Roche, Gerald Gui, Kelvin Ramsey, Paul Harris, Stuart James, Kieran Power, Shelley Potter, Richard Sutton, Jamie McIntosh, Nicola Laurence, Louise MacLennan, Robert Milligan, Henry Cain, Adam Critchley, Joe O’Donoghue, Loraine Kalra, Nick Collis, Gina Weston-Petrides, Roanne Fiddes, Victoria Brown, Anna Aertssen, Diana Slade-Sharman, Mansoor Khan, Caroline McGuiness, Vittoria Amorosi, Santanelli di Pompeo Fabio, Georgios Exarchos, Natasha Jiwa, Jennifer Hu, Serena Ledwidge, Laura Johnson, Anthony Peel, Naseem Dhooma, Eric Farrell, Liam Devane, Ruth Tevlin, Enda McDermott, Ruth Prichard, Denis Evoy, Jane Rothwell, James Geraghty, Colin Morrison, Catriona Lawlor, Fiona Langlands, Lauren Taylor, Philip Turton, Raj Achuthan, Kieran Horgan, Shireen Mckenzie, Brian Hogan, Mark Lansdown, Channegowda Navin, Liz Sherwin, Caroline Mortimer, Neeraj Garg, Rahma Adam, Tahera Arif, Zbigniew Kryjak, Deedar Ali, Ravi Sowdi, Elena Fage, Senthurun Mylvaganam, Pilar Matey, Raghavan Vidya, Tapan Sircar, Oubida Asaad, Pud Bhaskar, Matei Dordea, Ada Chrysafi, Damian McCartan, Rajiv Dave, Rachel Foster, Rebecca Wilson, Sylvia Okwemba, Yousef Majeed, Ciara O’Brien, Vinod Mathen, John Murphy, Nicola Barnes, Ashu Gandhi, James Harvey, Cliona C Kirwan, Richard Johnson, Krupali Patel, Maria Dalmau Ribas, Natali Vigneswaran, Tom Challoner, Joanna Skillman, Alan Park, Maged Rizkalla, Abigail Tomlins, Kat McEvoy, Sadaf Jafferbhoy, Soni Soumian, Sankaran Narayanan, Robert Kirby, Sladana Bajrusevic, Joseph Maalo, Michalis Charalambous, Lee Min Lai, Kelvin Chong, Simon Thomson, Sherif Monib, Leena Chagla, Riccardo Audisio, Rieka Taghizadeh, Azhar Iqbal, Karen James, Maria Callaghan, Shabbir Poonawala, Jonathan Lund, Raman Vinayagam, Sadaf Jafferbhoy, Steven Thrush, Rachel Bright Thomas, Michelle Mullan, Jevan Taylor, Ryo Yoshimura, Tom Mathew, Ben Mancey Jones, Kailas Munot, Rana Nasr, Jenny Piper, Deena El-Sharief, Mohammed Mustafa

**Affiliations:** 1 Department of Breast Surgery, The Royal Marsden NHS Foundation Trust, Downs Road, Sutton, Surrey, SM2 5PT UK; 2 Department of Cancer Studies, Clinical Sciences Building, University of Leicester, Leicester, LE2 2LX UK; 3 Nightingale Breast Unit, Manchester University NHS Foundation Trust, Southmoor Road, Manchester, M23 9LT UK; 4 Population Health Sciences, Bristol Medical School, 39 Whatley Road, Clifton, Bristol, BS8 2PS UK; 5 Department of Plastic Surgery, University Hospitals Coventry and Warwickshire NHS Trust, Clifford Bridge Road, Coventry, CV2 2DX UK; 6 Nuffield Department of Orthopaedics, Rheumatology and Musculoskeletal Sciences, University of Oxford, Nuffield Orthopaedic Centre, Windmill Road, Headington, Oxford, OX3 7HE UK; 7 Department of Surgery and Cancer, Imperial College London, London, UK; 8 Norfolk and Norwich Hospital, Norwich, UK; 9 Bristol Breast Care Centre, North Bristol NHS Trust, Southmead Road, Bristol, BS10 5NB UK; 10 Linda McCartney Centre, Royal Liverpool and Broadgreen University Hospital, Prescot Street, Liverpool, L7 8XP UK

**Keywords:** Surgical oncology, Outcomes research, Cancer

## Abstract

**Background:**

Immediate breast reconstruction (IBR) is routinely offered to improve quality-of-life for women requiring mastectomy, but there are concerns that more complex surgery may delay adjuvant oncological treatments and compromise long-term outcomes. High-quality evidence is lacking. The iBRA-2 study aimed to investigate the impact of IBR on time to adjuvant therapy.

**Methods:**

Consecutive women undergoing mastectomy ± IBR for breast cancer July–December, 2016 were included. Patient demographics, operative, oncological and complication data were collected. Time from last definitive cancer surgery to first adjuvant treatment for patients undergoing mastectomy ± IBR were compared and risk factors associated with delays explored.

**Results:**

A total of 2540 patients were recruited from 76 centres; 1008 (39.7%) underwent IBR (implant-only [*n* = 675, 26.6%]; pedicled flaps [*n* = 105,4.1%] and free-flaps [*n* = 228, 8.9%]). Complications requiring re-admission or re-operation were significantly more common in patients undergoing IBR than those receiving mastectomy. Adjuvant chemotherapy or radiotherapy was required by 1235 (48.6%) patients. No clinically significant differences were seen in time to adjuvant therapy between patient groups but major complications irrespective of surgery received were significantly associated with treatment delays.

**Conclusions:**

IBR does not result in clinically significant delays to adjuvant therapy, but post-operative complications are associated with treatment delays. Strategies to minimise complications, including careful patient selection, are required to improve outcomes for patients.

## Background

Breast cancer is the most common female cancer worldwide with 1.7 million new cases diagnosed each year.^1^ Despite improvements in treatment, however, mastectomy remains the primary surgical treatment for almost 40% of women^2,3^ and immediate breast reconstruction (IBR) is offered with the aim of improving quality-of-life.^4^

Although psychosocial outcomes are an important consideration when planning treatment, oncological safety remains paramount. Breast reconstruction is associated with more complications than simple mastectomy,^5^ and concerns have been raised that the increased complication rate may lead to the delay or omission of adjuvant chemotherapy or radiotherapy,^6^ which may compromise oncological outcomes. The clinical significance of short delays is unclear, but two recent large population-based studies have shown that patients experiencing delays of more than 90 days in the delivery of chemotherapy experienced worse overall and cancer-specific survival.^7,8^ Furthermore, a recent meta-analysis suggests a 15% decrease in overall survival for every four-week delay in the delivery of adjuvant chemotherapy.^9^ Delays to radiotherapy have similarly adverse effects but the time-frames are less well-established. A meta-analysis including 21 retrospective breast cancer studies suggested an increased risk of loco-regional recurrence if radiotherapy was delayed by more than 8 weeks following surgery,^10^ but other large cohort studies have demonstrated no deleterious effects with delays of up to 20 weeks.^11^

Evidence regarding the impact of IBR on the delivery of adjuvant therapy, however, is inconsistent.^6^ A recent systematic review^6^ failed to demonstrate a clinically significant delay in the initiation of chemotherapy but included 14 mainly single-centre studies with significant heterogeneity and these results cannot be relied upon. Two large population-based studies, however have recently reported delays to the start of chemotherapy in the patients undergoing IBR. One study did not differentiate between types of breast reconstruction^7^ and the second used patients undergoing breast conserving surgery as a control group and demonstrated delays in patients undergoing mastectomy without reconstruction as well as those undergoing immediate autologous reconstruction procedures^8^ making these findings difficult to interpret.

High-quality evidence regarding the impact of IBR on the delivery of adjuvant therapy compared with mastectomy alone is therefore lacking. Randomised trials (RCTs) provide the best evidence of treatment effect but are inappropriate in this context. A large-scale prospective cohort study is therefore required to generate high-quality data to allow patients and surgeons to make more informed decisions about potential treatment options. The trainee research collaborative model has recently emerged as a time and cost-effective method for delivering large-scale prospective studies in reconstructive breast surgery.^12^ This network of breast and plastic surgeons was utilised to deliver the iBRA-2 study to determine the impact of IBR on the delivery of adjuvant treatment.^13^

## Methods

### Study design and participants

A prospective multicentre cohort study was used to determine whether IBR influenced time to delivery of adjuvant therapy compared to mastectomy alone.

All breast or plastic surgical units performing mastectomy with or without IBR were invited to participate through the UK Trainee Collaborative Research Network (the Mammary Fold Academic and Research Collaborative and the Reconstructive Surgery Trials Network) and the UK professional associations (Association of Breast Surgery [ABS] and the British Association of Plastic Reconstructive and Aesthetic Surgeons [BAPRAS]).

Consecutive women aged 18 or over undergoing mastectomy with or without IBR using any technique for invasive or pre-invasive (ductal carcinoma in situ, DCIS) breast cancer with curative intent at participating centres between 1st July and 31st December 2016 were recruited to the study. Excluded were patients undergoing risk-reducing surgery (without a therapeutic mastectomy for breast cancer), partial mastectomy including wide local excision with volume replacement (latissimus dorsi mini-flaps; lateral intercostal perforator (LICAP) or thoracodorsal artery perforator (TDAP) flaps) or displacement techniques (therapeutic mammaplasty), and those with distant metastatic disease.

This study was classified as service evaluation by the UK National Health Service Research Authority Decision Tool (http://www.hra-decisiontools.org.uk/research/index.html), hence individual patient consent was not required. Each participating centre registered the study and obtained local clinical governance approvals before commencing patient recruitment. The study protocol was published in 2016.^13^

### Procedures

Patients were identified prospectively from clinics, multidisciplinary team (MDT) meetings and operating theatre lists. Simple demographic, comorbidity, operative and oncology data were collected for each participant. Decisions regarding the recommendation for adjuvant treatment were identified from the post-operative MDT meeting.

For patients in whom adjuvant therapy was recommended, data were collected on whether the offer was accepted and in patients electing to receive adjuvant therapy, date of the first treatment was recorded.

Data regarding post-operative complications were collected prospectively until the patient commenced adjuvant therapy or it was decided that adjuvant therapy would be omitted due to post-operative complications. Preliminary work suggested that adjuvant therapy was unlikely to commence earlier than 6 weeks post-operatively. Data collection in patients not requiring adjuvant treatment therefore continued from the last definitive cancer surgery until 6 weeks following surgery either by clinical assessment or note-review in those not attending for follow-up.

The REDCap electronic data-capture system^14^ (http://www.projectredcap.org/) was used in data collection.

The study processes were piloted over a 4-week period to ensure the feasibility of the study and to refine the case report forms before commencing national recruitment.

For the purposes of the analysis, patients were categorised into four groups according to the most complex procedure received as: (i) mastectomy only without reconstruction; (ii) mastectomy and IBR with implant-only techniques; (iii) mastectomy and IBR with pedicled flaps and (iv) mastectomy and IBR with free-flap techniques. Implant-based procedures included any reconstruction in which only expanders/implants were used to reconstruct the breast. This included one or two-stage procedures with or without biological (e.g. acellular dermal matrix) or synthetic (e.g. titanium-coated polypropylene) mesh irrespective of whether the implant/expander was placed in a pre- or subpectoral position. Pedicled flap procedures included any pedicled flap used to reconstruct the breast with or without an implant/expander and included latissimus dorsi (LD) and transverse rectus abdominus myocutaneous (TRAM) flaps. Free-flap procedures included any technique in which a microvascular free-flap was used for IBR and included deep inferior epigastric perforator (DIEP), superficial inferior epigastric perforator (SIEA), superior and inferior gluteal artery perforator (SGAP and IGAP) and transverse upper gracillis, (TUG) procedures.

### Outcome measures

The primary outcome was time in days from last definitive cancer surgery to the first adjuvant treatment. The last definitive cancer surgery included any additional procedures recommended by the MDT for oncological reasons (e.g. axillary clearance) but did not include any surgery for post-operative complications (e.g. debridement of skin-flap necrosis). First adjuvant therapy was defined as the first dose of chemotherapy or the first fraction of radiotherapy. Time to endocrine therapy was not included. In patients for whom more than one modality of adjuvant treatment was recommended, only the start date for the first adjuvant therapy was recorded. Significant treatment delays to (i) chemotherapy and (ii) radiotherapy were defined based on the best available evidence^7,8,10^ as delays of >90 days for chemotherapy^7,8^ and >8 weeks for radiotherapy.^10^

Secondary outcomes included post-operative complications, re-admission to hospital following discharge and unplanned re-operation for complications within 6 weeks of the last definitive cancer surgery or prior to the start of adjuvant therapy. All complications were defined a priori. Major complications were defined as any complication requiring re-admission or re-operation. Minor complications were defined as those managed conservatively.^13^

### Quality assurance

For quality assurance (QA) purposes, the principal investigator at each participating site was asked to independently validate 5–10% of the submitted data for each unit and to check complete case ascertainment. If concordance between the data entered on REDCap and that independently validated was <90%, the unit’s data were excluded from the analysis consistent with the QA procedure used in other collaborative projects.^13^

### Statistical analysis

Descriptive summary statistics were calculated for each variable for the cohort overall and split by operative procedure. Categorical data were summarised by counts and percentages. Continuous data were summarised by median, interquartile range (IQR) and range. Procedure groups were compared using appropriate non-parametric statistics. Complications and oncological outcomes were summarised by procedure and by patient.

Univariable and multivariable logistic regression analysis was used to explore clinico-pathological variables hypothesised to be associated with the development of (i) any complication and (ii) major complications, as these were considered most likely to impact on time to adjuvant therapy. Variables of interest were defined a priori based on the literature and expert opinion and included patient and procedure-related variables, namely age, smoking, body mass index (BMI), diabetes, ischaemic heart disease (IHD), other comorbidities, previous surgery and/or radiotherapy to the ipsilateral breast; neoadjuvant chemotherapy (NAC), American Society of Anesthesiologists (ASA) grade, unilateral vs bilateral surgery, type of axillary surgery (none, sentinel node biopsy [SNB] or axillary node clearance [ANC]) and procedure type (mastectomy, implant-based, pedicled or free-flap reconstruction).

Time from surgery to first adjuvant therapy was calculated for all patients and for those undergoing (i) chemotherapy and (ii) radiotherapy as their first adjuvant treatment separately in each procedure group, with adjuvant therapy as the event. This analysis was repeated stratifying by whether the patient had no, minor, or major complications. Kaplan–Meier analyses, univariable and multivariable Cox survival models of time to first adjuvant therapy and time to (i) chemotherapy and (ii) radiotherapy separately split by procedure type were created, including patient age, BMI, diabetes, IHD, other comorbidities, smoking, ASA grade, unilateral vs bilateral surgery, procedure type and the presence of complications (none, minor or major) as variables of interest, clustered by centre. The Kaplan–Meier graphs of time to adjuvant therapy were curtailed at 150 days, when only 10 patients remain in follow-up, to better focus on the majority of patients.

STATA 15 (STATA, Inc., Texas) was used for all analyses.

## Results

In total, 2652 patients were recruited to the study from 76 centres across the UK (*n* = 66), Europe (*n* = 9) and North Africa (*n* = 1). Of these, 112 (4.4%) were excluded; 19 (0.7%) had surgery outside of the study period; 55 (2.1%) had risk-reducing surgery only; 6 (0.2%) did not undergo a mastectomy and 24 (0.9%) had incorrect or important missing data (e.g. operation date or procedure type). Eight (0.3%) patients had ‘other’ forms of reconstruction. These could not be appropriately categorised, hence were excluded. 2540 patients were therefore included in the analysis. Of these, 1008 (39.7%) underwent IBR with implant-based (*n* = 675), pedicled flaps (*n* = 105) or free-flap (*n* = 228) techniques.

### Patient demographics

Patient demographics are summarised in Table 1. Women undergoing IBR were younger and had fewer comorbidities than patients undergoing mastectomy only. More patients undergoing IBR received NAC than those undergoing simple mastectomy and patients undergoing IBR were more likely to have undergone an up-front SNB before their reconstruction, particularly if they were undergoing tissue-based procedures. Bilateral surgery for risk reduction or symmetry was more common in patients undergoing implant-based or free-flap reconstruction (Table 1).

**Table 1:** Demographics of participants in the iBRA-2 study by procedure type

	All patients (*n* = 2540)	Mastectomy only (*n* = 1532, 60.3%)	Implant (*n* = 675, 26.6%)	Pedicled flap (*n* = 105, 4.1%)	Free-flap (*n* = 228, 8.9%)	*P*-value
*Age (years): median (IQR) (range)*	58 (48–69) (21–96)	65 (54–75) (26–96)	50 (43–57) (23–82)	52 (47–60) (25–74)	50 (44.5–56) (21–72)	<0.001^a^
<35	89 (3.5)	34 (2.2)	42 (6.2)	4 (3.8)	9 (4.0)	
35–44	337 (13.3)	115 (7.5)	160 (23.7)	14 (13.3)	48 (21.1)	
45–54	655 (25.8)	257 (16.8)	248 (36.7)	50 (47.6)	100 (43.9)	
55–64	537 (21.1)	320 (20.9)	141 (20.9)	22 (21.0)	54 (23.7)	
65–75	509 (20.0)	406 (26.5)	71 (10.5)	15 (14.3)	17 (7.5)	
>75	402 (15.8)	392 (25.6)	10 (1.5)	0 (0.0)	0 (0.0)	
Not reported	11 (0.4)	8 (0.5)	3 (0.4)	0 (0.0)	0 (0.0)	
*BMI (median) kg/m* ^*2*^	26.4	27.3	24.4	26.6	27.4	<0.001^a^
(IQR) (range)	(23.2–30.7) (13.4–80.7)	(23.7–32.2) (13.4–80.7)	(21.9–27.6) (16.0–61.4)	(23.3–30.6) (18.5–39.2)	(24.2–30.1) (15.6–31.1)	
Underweight	55 (2.2)	33 (2.2)	20 (3.0)	0 (0.0)	2 (0.9)	
Normal	880 (34.7)	445 (29.0)	328 (48.6)	37 (35.2)	70 (30.7)	
Overweight	769 (30.3)	457 (29.8)	191 (28.3)	35 (33.3)	86 (37.7)	
Obese	380 (15.0)	252 (16.4)	65 (9.6)	22 (21.0)	41 (18. 0)	
Severely obese	277 (10.9)	221 (14.4)	35 (5.2)	5 (4.8)	16 (7.0)	
Not reported	179 (7.1)	124 (8.1)	36 (5.3)	6 (5.7)	13 (5.7)	
*Smoking status*						0.015^b^
Non-smoker	1829 (71.6)	1082 (70.6)	499 (73.9)	75 (71.4)	163 (71.5)	
Current smoker	276 (10.9)	180 (11.7)	73 (10.8)	12 (11.4)	11 (4.8)	
Ex-smoker	401 (15.8)	241 (15.7)	91 (13.5)	18 (17.1)	51 (22.4)	
Missing	44 (1.7)	29 (1.9)	12 (1.8)	0 (0.0)	3 (1.3)	
*Comorbidities*						
Diabetes	232 (9.1)	189 (12.3)	25 (3.7)	7 (6.7)	11 (4.8)	<0.001^b^
Ischaemic heart disease	140 (5.5)	133 (8.7)	3 (0.4)	2 (1.9)	2 (0.9)	<0.001^b^
Other comorbidity	1186 (46.7)	848 (55.3)	222 (32.9)	36 (34.3)	80 (35.1)	<0.001^b^
*Previous oncological therapy*						
Radiotherapy to ipsilateral breast	240 (9.5)	158 (10.3)	40 (5.9)	16 (15.2)	26 (11.4)	0.011^b^
Neoadjuvant chemotherapy	422 (16.6)	230 (15.0)	128 (19.0)	21 (20.0)	43 (18.9)	0.001^b^
Neoadjuvant endocrine therapy	186 (7.3)	136 (8.9)	28 (4.1)	8 (7.6)	14 (6.1)	<0.001^b^
*Previous surgery to ipsilateral breast*						
Any surgery	546 (21.5)	299 (19.5)	147 (21.8)	37 (35.2)	63 (27.6)	0.001^b^
Cosmetic surgery	32 (1.3)	7 (0.5)	17 (2.5)	1 (1.0)	7 (3.1)	<0.001^b^
Oncological surgery	477 (18.7)	271 (17.7)	119 (17.6)	33 (31.4)	54 (23.7)	0.001^b^
*Previous surgery to ipsilateral axilla*						
Any axillary surgery	502 (19.8)	230 (15.0)	148 (21.9)	40 (38.1)	84 (36.8)	<0.001^b^
Axillary clearance	102 (4.0)	70 (4.6)	15 (2.2)	10 (9.5)	7 (3.1)	<0.001^b^
Axillary sample	41 (1.6)	30 (2.0)	2 (0.3)	2 (1.9)	7 (3.1)	
SNB (with BCS)	192 (7.6)	107 (7.0)	54 (8.0)	11 (10.5)	20 (8.8)	
Stand-alone SNB	167 (6.6)	23 (1.5)	77 (11.4)	17 (16.2)	50 (21.9)	
*ASA grade*						<0.001^b^
Grade 1	705 (27.8)	333 (21.7)	273 (40.4)	40 (38.1)	59 (25.9)	
Grade 2	1506 (59.3)	906 (59.1)	379 (56.2)	61 (58.1)	160 (70.2)	
Grade 3	313 (12.3)	279 (18.2)	23 (3.4)	3 (2.9)	8 (3.5)	
Grade 4	6 (0.2)	6 (0.4)	0 (0.0)	0 (0.0)	0 (0.0)	
Missing	10 (0.4)	8 (0.5)	0 (0.0)	1 (1.0)	1 (0.4)	
*Laterality of surgery*						<0.001^b^
Unilateral Mx ± BR	2235 (88.0)	1427 (93.2)	528 (78.2)	96 (91.4)	184 (80.7)	
Bilateral Mx ± BR	189 (7.4)	71 (4.6)	98 (14.5)	1 (1.0)	19 (8.3)	
Unilateral procedure + contralateral symmetrisation	91 (3.6)	19 (1.2)	43 (6.4)	8 (7.6)	21 (9.2)	
Unilateral procedure + contralateral oncological procedure	25 (1.0)	15 (1.0)	6 (0.9)	0 (0.0)	4 (1.8)	
*Indication for bilateral surgery* (*n* *=* *305*)						<0.001^b^
Bilateral malignancy	82 (26.9)	39 (37.1)	36 (24.5)	0 (0.0)	7 (15.9)	
Unilateral malignancy/contralateral risk reduction	116 (38.0)	35 (33.3)	66 (44.9)	2 (22.2)	13 (29.6)	
Unilateral malignancy/contralateral symmetrisation^c^	93 (30.5)	20 (19.1)	42 (28.6)	7 (77.8)	24 (54.6)	
Unilateral malignancy/other	14 (4.6)	11 (10.5)	3 (2.0)	0 (0.0)	0 (0.0)	

*ASA* American Society of Anaesthesiologists, *BCS* breast conserving surgery, *BMI* body mass index, *BR* breast reconstruction, *IQR* interquartile range, *Mx* mastectomy, *SNB* sentinel node biopsy

^a^Kruskal–Wallis test

^b^Chi-squared test

^c^Includes simple mastectomy/reduction mammoplasty/mastopexy/augmentation and contralateral and reconstruction

### Post-operative complications

The 2540 patients underwent 2732 procedures including 773 implant-based reconstructions (157 subpectoral expanders; 410 subpectoral reconstructions with biological or synthetic mesh; 105 dermal-sling procedures and 98 prepectoral reconstructions), 106 pedicled flaps (62 autologous LD, 39 LD with implant, 2 pedicled TRAM and 2 other) and 247 free-flap procedures (219 DIEPs, 16 free TRAMs, 4 SIEA, 7 TUG flaps and 1 other). Details of complications by procedure are summarised in supplementary table 1.

Overall, 929 (36.6%) of patients in the study experienced at least one post-operative complication (Table 2). Univariable analysis identified age, BMI, IHD, diabetes, having other comorbidities, smoking, ASA grade and undergoing an ANC but not IBR as risk factors associated with developing a post-operative complication (Table 2). Age, BMI, having other comorbidities, smoking and undergoing an ANC remained strongly associated with post-operative complications in the multivariable model, whereas undergoing bilateral surgery and free-flap reconstruction were also identified as independent risk factors for complications in the multivariable analysis.

**Table 2:** Univariable and multivariable logistic regression for any post-operative complication and major complications

	Any complication					Major complications				
	Univariable			Multivariable (*n* = 2191)		Univariable			Multivariable (*n* = 2206)	
	*N* (events, %)	Odds ratio (95% confidence intervals)	*P*-value (95% confidence intervals)	Odds ratio	*P*-value	*N* (events, %)	Odds ratio (95% confidence intervals)	*P*-value	Odds ratio (95% confidence intervals)	*P*-value
*Procedure type*	2517 (929, 37%)					2540 (221, 9%)				
Mastectomy only	1517 (570, 38%)	Reference		Reference		1532 (76, 5%)	Reference		Reference	
Implant-based	667 (223, 33%)	0.83 (0.64, 1.08)	0.171	1.19 (0.85, 1.67)	0.301	675 (100, 15%)	3.33 (2.18, 5.09)	<0.001	4.34 (2.35, 7.99)	<0.001
Pedicled flap	105 (42, 40%)	1.11 (0.61, 2.00)	0.735	1.42 (0.70, 2.87)	0.333	105 (7, 7%)	1.37 (0.66, 2.83)	0.398	1.52 (0.74, 3.13)	0.257
Free-flap	228 (94, 41%)	1.17 (0.83, 1.64)	0.377	1.66 (1.07, 2.57)	0.023	228 (38, 17%)	3.83 (2.41, 6.07)	<0.001	4.88 (2.63, 9.04)	<0.001
Age	2506 (926, 37%)	1.01 (1.01, 1.02)	<0.001	1.01 (1.00, 1.02)	0.003	2529 (221, 9%)	0.99 (0.98, 1.00)	0.006	1.01 (0.99, 1.02)	0.451
*BMI*	2339 (869, 37%)					2361 (208, 9%)				
Underweight	54 (15, 28%)	0.90 (0.48, 1.68)	0.742	0.85 (0.44, 1.62)	0.614	55 (4, 7%)	0.88 (0.24, 3.17)	0.845	0.88 (0.23, 3.43)	0.856
Normal weight	872 (261, 30%)	Reference		Reference		880 (72, 8%)	Reference		Reference	
Overweight	760 (297, 39%)	1.50 (1.21, 1.86)	<0.001	1.41 (1.11, 1.79)	0.005	769 (57, 7%)	0.90 (0.63, 1.28)	0.553	0.98 (0.68, 1.43)	0.926
Obese	379 (169, 45%)	1.88 (1.47, 2.41)	<0.001	1.72 (1.31, 2.26)	<0.001	380 (46, 12%)	1.55 (1.09, 2.19)	0.015	1.99 (1.37, 2.89)	<0.001
Severely obese	274 (127, 46%)	2.02 (1.47, 2.79)	<0.001	1.70 (1.16, 2.50)	0.007	277 (29, 10%)	1.31 (0.86, 2.00)	0.206	1.69 (1.00, 2.84)	0.049
*Comorbidities*										
IHD	2494 (922, 37%)					2515 (220, 9%)				
No	2356 (859, 36%)	Reference		Reference		2375 (212, 9%)	Reference		Reference	
Yes	138 (63, 46%)	1.46 (1.03, 2.07)	0.031	1.08 (0.71, 1.64)	0.724	140 (8, 6%)	0.62 (0.33, 1.67)	0.138	0.86 (0.37, 2.01)	0.728
Diabetes	2457 (907, 37%)					2479 (216, 9%)				
No	2227 (797, 36%)	Reference		Reference		2247 (191, 9%)	Reference		Reference	
Yes	230 (110, 48%)	1.64 (1.28, 2.11)	<0.001	1.15 (0.84, 1.57)	0.396	232 (25, 11%)	1.30 (0.83, 2.04)	0.253	1.53 (0.95, 2.48)	0.081
Other	2500 (925, 37%)					2522 (220, 9%)				
No	1319 (4200, 32%)	Reference		Reference		1336 (105, 8%)	Reference		Reference	
Yes	1181 (505, 43%)	1.60 (1.29, 1.98)	<0.001	1.36 (1.06, 1.73)	0.014	1186 (115, 10%)	1.26 (0.98, 1.62)	0.074	1.52 (1.13, 2.04)	0.006
*Smoking status*	2474 (915, 37%)					2496 (220, 9%)				
Non-smoker	1800 (630, 35%)	Reference		Reference		1819 (147, 8%)	Reference		Reference	
Ex-smoker	399 (178, 45%)	1.50 (1.20, 1.86)	<0.001	1.42 (1.10, 1.82)	0.006	401 (40, 10%)	1.26 (0.85, 1.87)	0.253	1.27 (0.84, 1.94)	0.262
Current smoker	275 (107, 39%)	1.18 (0.93, 1.51)	0.18	1.36 (1.05, 1.77)	0.019	276 (33, 12%)	1.54 (1.01, 2.35)	0.042	2.04 (1.33, 3.12)	0.001
*Previous surgery to ipsilateral breast*	2511 (927, 37%)					2534 (221, 9%)				
No	1968 (728, 37%)	Reference		Reference		1988 (170, 9%)	Reference		Reference	
Yes	543 (199, 37%)	0.99 (0.81, 1.20)	0.885	1.06 (0.77, 1.45)	0.74	546 (51, 9%)	1.10 (0.76, 1.59)	0.604	0.98 (0.58, 1.65)	0.938
*Previous radiotherapy*	2498 (924, 37%)					2521 (221, 9%)				
No	2258 (834, 37%)	Reference		Reference		240 (23, 10%)	Reference		Reference	
Yes	240 (90, 38%)	1.02 (0.79, 1.33)	0.858	0.98 (0.69, 1.39)	0.903	2281 (198, 9%)	1.12 (0.70, 1.77)	0.642	1.24 (0.63, 2.44)	0.534
*Neoadjuvant chemotherapy*	2497 (923, 37%)					2520 (220, 9%)				
No	2078 (782, 38%)	Reference		Reference		422 (40, 9%)	Reference		Reference	
Yes	419 (141, 34%)	0.84 (0.64, 1.10)	0.203	0.86 (0.64, 1.14)	0.292	2098 (180, 9%)	1.12 (0.71, 1.76)	0.638	1.24 (0.79, 1.95)	0.35
*ASA grade*	2507 (926, 37%)					2530 (220, 9%)				
1	702 (214, 30%)	Reference		Reference		705 (60, 9%)	Reference		Reference	
2	1487 (571, 38%)	1.42 (1.15, 1.74)	0.001	1.00 (0.78, 1.28)	0.996	1506 (136, 9%)	1.07 (0.79, 1.44)	0.668	0.89 (0.62, 1.28)	0.526
3	312 (138, 44%)	1.81 (1.37, 2.39)	<0.001	0.99 (0.66, 1.48)	0.948	313 (24, 8%)	0.89 (0.54, 1.47)	0.654	0.88 (0.45, 1.72)	0.711
4	6 (3, 50%)	2.28 (0.45, 11.53)	0.319	0.91 (0.15, 5.59)	0.917	6 (0, 0%)	NA	—	NA	—
*Bilateral surgery*	2517 (929, 37%)					2540 (221, 9%)				
No	2214 (803, 36%)	Reference		Reference		2235 (175, 8%)	Reference		Reference	
Yes	303 (126, 42%)	1.25 (0.94, 1.69)	0.128	1.47 (1.08, 2.00)	0.015	305 (46, 15%)	2.09 (1.55, 2.82)	<0.001	1.52 (1.08, 2.14)	0.018
*Axillary surgery*	2517 (929, 37%)					2540 (221, 9%)				
None	416 (136, 33%)	Reference		Reference		418 (41, 10%)	Reference		Reference	
Sentinel node biopsy/Axillary sample	1410 (499, 35%)	1.13 (0.91, 1.39)	0.265	1.23 (0.92, 1.63)	0.156	1423 (132, 9%)	0.94 (0.64, 1.38)	0.754	1.10 (0.69, 1.75)	0.696
Axillary clearance	691 (294, 43%)	1.52 (1.17, 1.98)	0.002	1.78 (1.29, 2.45)	<0.001	699 (48, 7%)	0.68 (0.43, 1.07)	0.093	0.81 (0.53, 1.23)	0.324

*IHD* Ischaemic heart disease

Major complications which required re-admission to hospital or further surgery (Table 2) were experienced by 221 (8.7%) of patients. Implant-based and free-flap reconstruction, age, BMI, smoking and bilateral surgery were associated with major complications in the univariable analysis. All of these variables except for age, remained strongly associated with major complications in the multivariable model but implant-based (adjusted odds ratio [aOR] 4.34, 95% confidence interval [CI] 2.35–7.99) and free-flap reconstruction (aOR 4.88, 95% CI 2.63–9.04) were the strongest predictors for major complications in this analysis (Table 2).

### Adjuvant treatment recommendations and time to adjuvant therapy

Table 3 summarises the post-operative pathology for the 2607 mastectomies performed for oncological indications. IBR was more likely to be performed following mastectomy for extensive DCIS or multifocal disease and in node-negative patients than simple mastectomy resulting in fewer patients in the IBR group requiring adjuvant chemotherapy or radiotherapy.

**Table 3:** Post-operative histology in procedures performed for malignancy

	All procedures performed for cancer (*n* = 2607)	Mastectomy only (*n* = 1564)	Implant (*n* = 707)	Pedicled flap (*n* = 105)	Free-flap (*n* = 231)	*P*-value
Patients having NAC with a complete pathological response (*n* = 408)	135 (32.0)	66 (29.1)	52 (41.9)	9 (42.9)	8 (22.2)	0.031
*Invasive status*						<0.001
Pre-invasive disease	388 (14.8)	141 (9.0)	163 (23.1)	26 (24.8)	58 (25.1)	
Invasive disease	2186 (83.9)	1413 (90.4)	533 (75.4)	77 (73.3)	163 (70.6)	
Not reported	33 (1.3)	10 (0.36)	11 (1.6)	2 (1.9)	10 (4.3)	
*Focality*						0.001
Unifocal disease	1740 (66.7)	1091 (69.8)	446 (63.1)	72 (68.6)	131 (56.7)	
Multifocal disease	836 (32.1)	455 (29.1)	251 (35.5)	33 (31.4)	97 (42.0)	
Not reported	31 (1.2)	18 (1.2)	10 (1.4)	0 (0.0)	3 (1.3)	
*Invasive disease (n* *=* *2186) grade*						0.045
Grade 1	179 (8.2)	98 (6.9)	58 (10.9)	7 (9.1)	16 (9.8)	
Grade 2	1187 (54.3)	759 (53.7)	285 (53.5)	47 (61.0)	96 (58.9)	
Grade 3	800 (36.6)	543 (38.4)	186 (24.1)	21 (27.3)	50 (30.7)	
Not reported	20 (0.9)	13 (0.9)	4 (0.8)	2 (2.6)	1 (0.6)	
*Histological type*						0.489
Ductal	1540 (70.5)	986 (69.8)	382 (71.7)	55 (71.4)	117 (71.8)	
Lobular	373 (17.1)	246 (17.4)	89 (16.7)	10 (13.0)	28 (17.2)	
Mixed	121 (5.5)	80 (5.7)	26 (4.9)	4 (5.2)	11 (6.8)	
Other	141 (6.5)	95 (6.7)	34 (6.4)	6 (7.8)	6 (3.7)	
Not reported	11 (0.5)	6 (0.4)	2 (0.4)	2 (2.6)	1 (0.6)	
*Tumour stage*						<0.001
Tis	388 (14.9)	141 (9.0)	163 (23.1)	26 (24.8)	58 (25.1)	
T1a (<0.5 cm)	187 (7.2)	88 (5.6)	71 (10.0)	9 (8.6)	19 (8.2)	
T1b (0.5–1 cm)	179 (6.9)	89 (5.7)	63 (8.9)	13 (12.4)	14 (6.1)	
T1c (1–2 cm)	578 (22.2)	359 (23.0)	156 (22.1)	17 (16.2)	46 (19.9)	
T2 (2–5 cm)	948 (36.4)	672 (43.0)	185 (26.2)	28 (26.7)	63 (27.3)	
T3 (>5 cm)	272 (10.4)	190 (12.2)	55 (7.8)	9 (8.6)	18 (7.8)	
Not reported	55 (2.1)	25 (1.6)	14 (2.0)	3 (2.9)	13 (5.6)	
Lymphovascular invasion	637 (29.1)	435 (30.8)	134 (25.1)	22 (28.6)	46 (28.2)	0.141
*ER*						<0.001
Positive	1738 (79.5)	1106 (78.3)	445 (83.5)	56 (72.7)	131 (80.4)	
Negative	433 (19.8)	298 (21.1)	86 (16.1)	18 (23.4)	31 (19.0)	
Unknown	15 (0.7)	4 (0.3)	2 (0.4)	3 (3.9)	1 (0.6)	
*HER-2*						0.871
Positive	422 (19.3)	273 (19.3)	109 (20.5)	12 (15.6)	28 (17.2)	
Negative	1686 (77.1)	1087 (76.9)	408 (76.6)	61 (79.2)	130 (79.8)	
Unknown	78 (3.6)	53 (3.8)	16 (3.0)	4 (5.2)	5 (3.1)	
*Nodal status*						<0.001
N0	1663 (63.8)	905 (57.9)	523 (74.0)	71 (67.6)	164 (71.0)	
N1	944 (36.2)	659 (42.1)	184 (26.0)	34 (32.4)	67 (29.0)	
*Pre-invasive disease (n* *=* *388)*						0.396
Low grade	27 (7.0)	7 (5.0)	12 (7.4)	1 (3.8)	7 (12.1)	
Intermediate grade	90 (23.2)	38 (27.0)	38 (23.3)	5 (19.2)	9 (15.5)	
High grade	269 (69.3)	95 (67.4)	112 (68.7)	20 (76.9)	42 (72.4)	
Not reported	2 (0.5)	1 (0.7)	1 (0.6)	0 (0.0)	0 (0.0)	

**Fig. 1:**
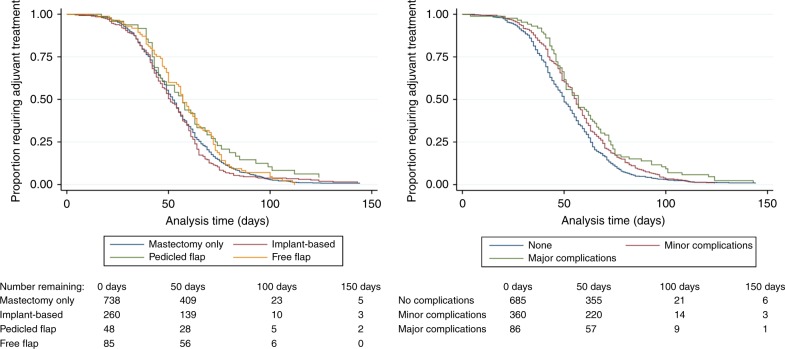
Kaplan–Meier analyses for time from last oncological surgery to first adjuvant treatment by **a** procedure type (left), **b** whether or not the patient developed post-operative complications (right)

Overall, 1235 (48.6%) patients were offered and accepted adjuvant treatment (Table 4). Time to adjuvant treatment differed between the groups, with those undergoing free-flap procedures having longer time to adjuvant therapy than those undergoing mastectomy only, adjusted hazard ratio (aHR) 0.84 (95% CI 0.71–0.99) (Table 5, Fig. 1a). The absolute differences between the median time to adjuvant treatment across the groups, however, were small; 52 (IQR 41–66) days for mastectomy only vs 57 (IQR 46–72) days for free-flap reconstruction (Table 5). The development of complications (Fig. 1b) and obesity were also associated with longer time to adjuvant therapy (Table 5). Median time to first chemotherapy was 47 days, (IQR 37–59). There were no significant differences in median time to chemotherapy or in the proportions of patients experiencing delays of greater that 90 days between the treatment groups (Table 4) but free-flap reconstruction (aHR 0.79, [95% CI 0.65–0.96]), major complications (aHR 0.72, [95% CI 0.54–0.94]) and obesity (aHR 0.75, [95% CI 0.57–0.99]) were associated with having longer time to chemotherapy in the multivariable model (Supplementary table 2). Median time to first fraction of radiotherapy was 60 days (IQR 48–73) with no differences in either the median time to radiotherapy or the proportion of patients experiencing significant treatment delays, defined as >8 weeks, between procedure types (Table 4). Major complications (aHR 0.70, [95% CI 0.53–0.93]) and smoking (aHR 0.73, [95% CI 0.57–0.94]) were associated with longer time to adjuvant radiotherapy in the multivariable model with older patients and those who had received neoadjuvant chemotherapy proceeding to radiotherapy more rapidly than other patient groups (Supplementary table 3).

**Table 4:** Multidisciplinary team (MDT) decision-making for adjuvant therapy

MDT decision-making per patient	All patients (*n* = 2540)	Mastectomy only (*n* = 1532)	Implant (*n* = 675)	Pedicled flap (*n* = 105)	Free-flap (*n* = 228)	*P*-value
*Chemotherapy*						
Recommended by MDT	649 (25.6)	421 (27.5)	154 (22.8)	28 (26.7)	46 (20.2)	<0.001
For discussion with patient	188 (7.4)	138 (9.0)	36 (5.3)	5 (4.8)	9 (3.9)	
For oncotype DX testing	181 (7.1)	95 (6.2)	64 (9.5)	5 (4.8)	17 (7.5)	
Not recommended by MDT	1509 (59.4)	872 (56.9)	415 (61.5)	67 (63.8)	155 (68.0)	
Not reported	13 (0.5)	6 (0.4)	6 (0.9)	0 (0.0)	1 (0.4)	
*Radiotherapy*						
Recommended by MDT	909 (35.7)	614 (40.1)	198 (29.3)	35 (33.3)	62 (27.2)	<0.001
For discussion with patient	125 (4.9)	86 (5.6)	19 (2.8)	1 (1.0)	19 (8.3)	
Not recommended by MDT	1492 (58.7)	828 (54.0)	449 (66.5)	69 (65.7)	146 (64.0)	
Not reported	14 (0.5)	4 (0.3)	9 (1.3)	0 (0.0)	1 (0.4)	
Patient accepts adjuvant treatment (either chemotherapy or radiotherapy or both)	1235 (48.6)	804 (52.5)	288 (42.7)	50 (47.6)	93 (40.8)	<0.001
Time from last oncological procedure to first adjuvant treatment (days) median (IQR) (*n* = 1131)	53 (41–65)	52 (41–66)	51 (41–63)	57 (42–73)	57 (46–72)	0.026
Chemotherapy as 1st adjuvant treatment	627 (55.4)	409 (55.4)	147 (56.5)	25 (52.1)	46 (54.1)	0.939
Time from last oncological procedure to 1st chemotherapy (days) median (IQR)	47 (37–59)	47 (37–59)	46 (35–57)	46 (39–58)	57 (41–70)	0.063
Reported delays of >90 days of planned chemotherapy (*n* = 637)	31 (4.9)	21 (5.1)	4 (2.7)	3 (11.5)	3 (6.5)	0.228
Radiotherapy as 1st adjuvant treatment	504 (44.6)	329 (44.6)	113 (43.5)	23 (47.9)	39 (45.9)	0.939
Time from last oncological procedure to 1st radiotherapy (days) median (IQR)	60 (48–73)	59 (48–73)	60 (45–68)	63 (53–85)	62 (50–76)	0.248
Reported delays of >56 days (8 weeks) of planned radiotherapy (*n* = 616)	389 (63.2)	258 (62.8)	83 (61.9)	23 (79.3)	25 (59.5)	0.308

**Table 5:** Cox univariable and multivariable survival analyses for time to adjuvant treatment

		Univariable		Multivariable (*N* = 1018)	
	*N* (%)	Hazard ratio^a^ (95% confidence intervals)	*P*-value	Hazard ratio^a^ (95% confidence intervals)	*P*-value
*Procedure type*	1131				
Mastectomy only	738 (65.3%)	Reference		Reference	
Implant-based	260 (23.0%)	1.08 (0.90, 1.29)	0.42	1.07 (0.88, 1.31)	0.496
Pedicled flap	48 (4.2%)	0.74 (0.49, 1.11)	0.149	0.72 (0.47, 1.08)	0.114
Free-flap	85 (7.5%)	0.84 (0.73, 0.97)	0.019	0.84 (0.71, 0.99)	0.036
*Post-operative complications*	1131				
None	685 (60.6%)	Reference		Reference	
Minor complications	360 (31.8%)	0.80 (0.70, 0.92)	0.002	0.85 (0.73, 1.00)	0.046
Major complications	86 (7.6%)	0.68 (0.54, 0.86)	0.001	0.63 (0.49, 0.82)	0.001
Chemotherapy as first adjuvant treatment	1131	1.79 (1.55, 2.06)	<0.001	2.42 (2.09, 2.81)	<0.001
Age	1128	1.00 (0.99, 1.00)	0.206	1.01 (1.00, 1.01)	0.058
*BMI*	1078				
Underweight	28 (2.6%)	1.03 (0.67, 1.59)	0.878	0.98 (0.66, 1.47)	0.933
Normal weight	387 (35.9%)	Reference		Reference	
Overweight	354 (32.8%)	0.99 (0.85, 1.15)	0.867	1.00 (0.85, 1.17)	0.953
Obese	188 (17.4%)	0.74 (0.65, 0.84)	<0.001	0.76 (0.64, 0.89)	0.001
Severely obese	121 (11.2%)	0.72 (0.61, 0.85)	<0.001	0.81 (0.67, 0.97)	0.023
*Comorbidities*					
Ischaemic heart disease	1128				
No	1079 (95.7%)	Reference		Reference	
Yes	49 (4.3%)	0.69 (0.53, 0.89)	0.005	0.82 (0.57, 1.18)	0.279
Diabetes	1103				
No	1002 (90.8%)	Reference		Reference	
Yes	101 (9.2%)	0.78 (0.68, 0.90)	0.001	0.94 (0.79, 1.13)	0.53
Other comorbidity	1123				
No	638 (56.8%)	Reference		Reference	
Yes	485 (43.2%)	0.88 (0.75, 1.03)	0.109	0.86 (0.70, 1.07)	0.17
*Smoking status*	1115				
Non-smoker	805 (72.2%)	Reference		Reference	
Ex-smoker	170 (15.3%)	1.11 (0.92, 1.34)	0.275	1.18 (0.96, 1.44)	0.111
Current smoker	140 (12.6%)	0.95 (0.81, 1.11)	0.496	0.88 (0.72, 1.07)	0.186
*Neoadjuvant chemotherapy*	1121				
No	829 (74.0%)	Reference		Reference	
Yes	292 (26.1%)	0.99 (0.87, 1.11)	0.808	1.71 (1.44, 2.03)	<0.001
*ASA grade*	1126				
1	357 (31.7%)	Reference		Reference	
2	654 (58.1%)	0.90 (0.78, 1.04)	0.154	1.09 (0.89, 1.33)	0.389
3	113 (10.0%)	0.85 (0.67, 1.08)	0.183	1.13 (0.78, 1.62)	0.524
4	2 (0.2%)	0.73 (0.61, 0.87)	0.001	1.37 (0.87, 2.14)	0.176
Bilateral surgery (vs none)	1131	0.92 (0.77, 1.10)	0.374	0.93 (0.75, 1.17)	0.546

*ASA* American Society of Anaesthesiologists, *BMI* body mass index

^a^aHR < 1 = increased time to adjuvant treatment aHR > 1 = shorter time to adjuvant treatment

Time to first adjuvant therapy (*P* < 0.001), time to chemotherapy (*P* < 0.001) and time to radiotherapy (*P* = 0.026), however all differed by whether the patient had no, minor or major complications, with an increasing trend seen across the three groups (no complications 50 days [IQR 39–63]; minor complications 56 days [IQR 42.5–69]; major complications 57 days [IQR 46–73], Supplementary table 4). Furthermore, patients experiencing complications were significantly more likely to experience significant treatment delays, defined as delays of >90 days for chemotherapy (*n* = 14, 3.6% of patients with no complications vs *n* = 7, 13% of patients with major complications; *P* = 0.011) and >8 weeks for radiotherapy (*n* = 222, 58.7% of patients with no complications vs *n* = 29, 70.7% of patients with major complications; *P* = 0.016, Supplementary table 4) than those whose procedures were uncomplicated.

## Discussion

Although free-flap reconstruction was associated with a longer time to adjuvant therapy than other procedure types, the absolute differences in time to treatment between the surgical groups is small. This study therefore suggests that IBR does not result in clinically significant delays in the delivery of adjuvant therapy compared to mastectomy alone. Complications, especially those requiring re-admission or further surgery however, are important and patients developing problems, irrespective of the procedure performed, were more likely to experience significant delays to both chemotherapy and radiotherapy in this analysis. The apparent paradox of no treatment delay despite the higher rate of major post-operative complications in the IBR group can be explained by careful patient selection for reconstructive surgery. Patients undergoing IBR were significantly younger and fitter, with fewer ‘risk factors’ for complications than patients undergoing mastectomy only and were less likely to require adjuvant treatment than the mastectomy only group. This is because IBR was more likely to be performed following mastectomy for extensive DCIS than for high-risk invasive disease with upfront axillary staging used to determine the likelihood that patients would require adjuvant treatment before their reconstructive procedure. This suggests that surgeons are cautious in offering IBR to patients likely to require adjuvant treatment.^15^ These concerns may reflect the impact of radiotherapy on the cosmetic outcome of reconstruction,^16^ but may also highlight anxiety about potential delays to adjuvant treatment with surgeons only opting to perform IBR in patients considered low risk. This study provides much-needed evidence to suggest that IBR does not lead to clinically significant delays in carefully selected low risk patient groups but does highlight that major complications can result in significant treatment delays. This study therefore strongly supports the need for careful patient selection to minimise complications and careful communication of the risks of post-operative problems and the potential oncological implication of complications on treatment delays with patients considering surgery. The higher risk of complications in patients undergoing bilateral surgery will particularly inform discussions with patients wishing to undergo simultaneous contralateral risk-reducing mastectomy and gives a sound rationale for delaying such surgery if adjuvant therapy is anticipated, particularly in implant-based reconstruction.

The findings of this study are consistent with other work suggesting that post-operative complications, rather than procedure type, are the main predictor of adjuvant treatment delays.^8^ This focuses attention on the need to reduce complications to improve outcomes for patients and is particularly relevant as reconstruction rates are increasing.^17^ Despite more procedures being performed, however, complications rates appear to be rising with re-operation for complications more than double that seen in the UK National Mastectomy and Breast Reconstruction Audit (NMBRA).^5^ This is a cause for concern as complications not only delay delivery of adjuvant treatments and but may also adversely impact long-term oncological outcomes by promoting a systemic inflammatory response.^18^ Implant-based procedures are now the most commonly-performed technique^19,20^ and although data from the NMBRA^5^ and the National Surgical Quality Improvement Program^21^ suggest implant reconstruction may be associated with fewer complications than other techniques, this study suggests that complications following implant-based and autologous reconstruction are broadly comparable. Reasons for this require further evaluation but may reflect the recent adoption of single-stage direct-to-implant mesh-assisted reconstruction in the UK,^22^ which may be associated with higher complication rates than the traditional two-stage procedures^23^ favoured in the US.^20^ Risk factors for complications, including smoking and high BMI are consistent with those previously reported^24,25^ and highlight the importance of careful patient selection if post-operative problems are to be avoided.

This is the first large prospective multicentre study to explore the impact of IBR on time to adjuvant therapy, but it has limitations. Firstly, this is an observational study and risk of bias must be considered. Consecutive patients undergoing mastectomy were recruited from participating centres but there were baseline differences in the treatment groups. Although it was possible to adjust for confounding factors such as age, BMI, smoking and ASA grade in the regression analyses, it is acknowledged that it is not possible to identify and control for all potential confounders which may have impacted the results. The study included patients from 76 centres across the UK and Europe and it is the largest study of its kind, but it is possible that participating units differed from those not taking part. However, this is unlikely, as almost half of all the breast and plastic surgical units in the UK elected to participate. A further consideration is that by only reporting delay to initiation of treatment, this study may underestimate both the overall complication rate of IBR and the true impact of reconstruction on the delivery of adjuvant therapy. This is particularly relevant for patients having implant reconstruction who may develop infection while receiving chemotherapy requiring treatment to be modified or stopped completely and the implant removed. Following patients during adjuvant treatment was not feasible with the trainee collaborative study design, but new collaborations with oncology trainees will allow these issues to be addressed in the future. Finally, this short-term study does not allow the long-term oncological impact of post-operative complications or any delays in the delivery of adjuvant therapy to be assessed. A data-linkage study to explore long-term oncological outcomes at 5 and 10 years is planned, allowing these important questions to be addressed. Therefore, although it is not possible to establish causality with an observational study design, RCTs in this setting are not possible and the iBRA-2 study provides much-needed evidence to support decision-making for IBR when adjuvant treatments may be needed.

The development of post-operative complications rather than the type of procedure performed has emerged as the key determinant of delays to the delivery adjuvant therapy in this study. Immediate implant-based and free-flap reconstructions, however, are associated with significantly higher rates of major complications than mastectomy alone and this is an important finding that should be fully discussed with patients considering reconstructive surgery. Avoiding IBR in high-risk patients including smokers and those with a high BMI and not performing unnecessary bilateral surgery may represent a simple strategy for reducing post-operative problems but this approach needs balanced against patients’ desire for IBR. Accurate and balanced communication of risks and benefits is a vital part of shared decision-making,^26^ and this study provides further evidence to inform this discussion. Major complications, irrespective of the procedure performed, result in delays to adjuvant treatment, hence strategies to minimise complications are needed for all patients undergoing breast cancer surgery to improve oncological outcomes,^18^ quality-of-life^27^ and minimise the overall cost of care.^28^ Standardising care may be one strategy by which outcomes may be improved and standardisation is the focus of the UK ‘Getting it Right First Time’ initiative. http://gettingitrightfirsttime.co.uk/surgical-specialty/breast-surgery/. Other strategies include altering treatment sequencing and routinely using neoadjuvant rather than adjuvant chemotherapy in patients electing to undergo IBR. This approach is safe, and these data show that those having neoadjuvant therapy start their adjuvant therapy sooner. It may also allow patients to address modifiable risk factors such as obesity or smoking before surgery although it is appreciated that these changes may be challenging. Increased use of neoadjuvant endocrine therapy may also have utility in high-risk groups. Neoadjuvant radiotherapy is a novel approach, which may provide an alternative treatment pathway in patients in whom radiotherapy is likely to be required.^29^ More accurately determining which patients may benefit from adjuvant therapy before the start of their breast cancer treatment, however may be the optimal solution and work to develop a more personalised approach using molecular markers and gene signatures is likely to reduce the number of future patients in whom adjuvant treatment may be indicated.^30,31^

IBR does not delay the delivery of adjuvant therapy, but implant-based and free-flap reconstructions are associated with higher rates of post-operative complications which are associated with treatment delays. Careful patient selection combined with accurate communication of risk are therefore vital if patients are to make fully informed decision about IBR when adjuvant therapy is likely to be needed. Further strategies to minimise the risk of complications such as increased use of neoadjuvant treatment may also be beneficial in this group. This study provides important information about the risk and impact of complications in IBR to help patients and surgeons make more informed decisions about their treatment options.

## Supplementary information

Supplementary table 1

Supplementary table 2

Supplementary table 3

Supplementary table 4

## Data Availability

The datasets generated during and/or analysed during the current study are not publicly available due to ongoing analyses but are available from the corresponding author on reasonable request.
