# Supplementary material for: The impact of immediate breast reconstruction on the time to delivery of adjuvant therapy: the iBRA-2 study
Source: Br J Cancer. 2019 Mar 29;120(9):883–95. doi: 10.1038/s41416-019-0438-1 (PMC6734656; doi:10.1038/s41416-019-0438-1)
Supplement: Supplementary file 1 — Supplementary table 1 [file 41416_2019_438_MOESM1_ESM.docx]

**Supplementary table 1: Detailed post-operative complications by procedure**

|  | **All procedures** | **Mastectomy only (n=1606)** | **Implant (n=773)** | **Pedicled flap (n=106)** | **Free flap (n=247)** |
| --- | --- | --- | --- | --- | --- |
|  | **(n=2732)** |  |  |  |  |
| **Seroma** |  |  |  |  |  |
| ***Mastectomy site/reconstructed breast*** |  |  |  |  |  |
| Requiring aspiration 1-2 times | 416 (15.2) | 342 (21.3) | 59 (7.6) | 6 (5.7) | 9 (3.6) |
| Requiring aspiration 3 or more times | 112 (4.1) | 92 (5.7) | 18 (2.3) | 1 (0.9) | 1 (0.4) |
| ***Donor site*** |  |  |  |  |  |
| Requiring aspiration 1-2 times | 24 (0.9) | NA | NA | 18 (17.0) | 6 (2.4) |
| Requiring aspiration 3 or more times | 5 (0.2) | NA | NA | 3 (2.8) | 2 (0.8) |
| **Haematoma** |  |  |  |  |  |
| ***Mastectomy site/reconstructed breast*** |  |  |  |  |  |
| Minor - Managed conservatively | 30 (1.1) | 21 (1.3) | 8 (1.0) | 0 (0.0) | 1 (0.4) |
| Major 1 - Requiring aspiration in clinic +/- US guidance | 19 (0.7) | 16 (1.0) | 3 (0.4) | 0 (0.0) | 0 (0.0) |
| Major 2 - Requiring evacuation in theatre | 54 (2.0) | 33 (2.1) | 16 (2.1) | 0 (0.0) | 5 (2.0) |
| **Donor site** |  |  |  |  |  |
| Minor - Managed conservatively | 1 (0.0) | NA | NA | 0 (0.0) | 1 (0.4) |
| Minor 1 - Requiring aspiration in clinic +/- US guidance | 1 (0.0) | NA | NA | 1 (0.9) | 0 (0.0) |
| Major 2 -Requiring evacuation in theatre | 4 (0.2) | NA | NA | 2 (1.9) | 2 (0.8) |
| **Wound infection** |  |  |  |  |  |
| ***Mastectomy site/reconstructed breast*** |  |  |  |  |  |
| Minor - Requiring oral antibiotics | 170 (6.2) | 110 (6.8) | 45 (5.8) | 7 (6.6) | 8 (3.2) |
| Major 1- Requiring admission for intravenous antibiotics | 59 (2.2) | 23 (1.4) | 25 (3.2) | 0 (0.0) | 11 (4.4) |
| Major 2 -Requiring surgical drainage/debridement | 49 (1.8) | 9 (0.6) | 34 (4.4) | 3 (2.8) | 3 (1.2) |
| **Donor site** |  |  |  |  |  |
| Minor - Requiring oral antibiotics | 8 (0.3) | NA | NA | 1 (0.9) | 7 (2.8) |
| Major 1- Requiring admission for intravenous antibiotics | 5 (0.2) | NA | NA | 1 (0.9) | 4 (1.6) |
| Major 2 -Requiring surgical drainage/debridement | 6 (0.2) | NA | NA | 0 (0.0) | 6 (2.4) |
| **Mastectomy skin-flap necrosis** |  |  |  |  |  |
| Minor - Managed conservatively | 63 (2.3) | 17 (1.1) | 24 (3.1) | 8 (7.5) | 14 (5.7) |
| Major 1 - Requiring debridement in clinic (no GA) | 13 (0.5) | 2 (0.1) | 7 (0.9) | 1 (0.9) | 3 (1.2) |
| Major 2 - Requiring surgical debridement in theatre | 31 (1.3) | 1 (0.1) | 24 (3.1) | 1 (0.9) | 5 (2.0) |
| **Donor site skin necrosis** |  |  |  |  |  |
| Minor - Managed conservatively | 5 (0.2) | NA | NA | 1 (0.9) | 4 (1.6) |
| Major 1 - Requiring debridement in clinic (no GA) | 2 (0.1) | NA | NA | 0 | 2 (0.8) |
| **Wound dehiscence** |  |  |  |  |  |
| ***Mastectomy site/reconstructed breast*** |  |  |  |  |  |
| Managed conservatively | 72 (2.6) | 35 (2.2) | 16 (2.1) | 2 (1.9) | 19 (7.7) |
| Requiring return to theatre for resuturing | 28 (1.0) | 3 (0.2) | 21 (2.7) | 1 (0.9) | 3 (1.2) |
| ***Donor site*** |  |  |  |  |  |
| Managed conservatively | 32 (1.2) | NA | NA | 5 (4.7) | 27 (10.9) |
| Requiring return to theatre for resuturing | 1 (0.0) | NA | NA | 0 (0.0) | 1 (0.4) |
| **Implant loss** | 53 (1.9) | NA | 53 (6.9) | 0 (0.0) | NA |
| **Impaired flap perfusion requiring return to theatre for exploration/revision (flap salvage)** | 5 (0.2) | NA | NA | 0 (0.0) | 5 (2.0) |
| **Flap necrosis** |  |  |  |  |  |
| Partial flap necrosis requiring surgical debridement | 7 (0.3) | NA | NA | 0 (0.0) | 7 (2.8) |
| Total flap necrosis requiring removal of flap | 3 (0.1) | NA | NA | 0 (0.0) | 3 (1.2) |
| **At least one breast or donor site complication** | 936 (34.3) | 570 (35.5) | 229 (29.6) | 43 (40.6) | 94 (38.1) |
| **Any major breast or donor site complication** | 222 (8.1) | 68 (4.2) | 110 (14.2) | 6 (5.7) | 38 (15.4) |

^a^Chi squared test, GA – general anaesthetic, US – ultrasound
