# Supplementary material for: The impact of immediate breast reconstruction on the time to delivery of adjuvant therapy: the iBRA-2 study
Source: Br J Cancer. 2019 Mar 29;120(9):883–95. doi: 10.1038/s41416-019-0438-1 (PMC6734656; doi:10.1038/s41416-019-0438-1)
Supplement: Supplementary file 2 — Supplementary table 2 [file 41416_2019_438_MOESM2_ESM.docx]

**Supplementary table 2: Cox univariable and multivariable survival analyses for time to chemotherapy**

|  |  | **Univariable** | | **Multivariable (N=584)** | |
| --- | --- | --- | --- | --- | --- |
|  |  | **Hazard Ratio^a^** |  | **Hazard Ratio^a^** |  |
|  | **N (%)** | **(95% Confidence Intervals)** | **P value** | **(95% Confidence Intervals)** | **P value** |
| **Procedure type** | **637** |  |  |  |  |
| Mastectomy only | 416 (65.3%) | Reference |  | Reference |  |
| Implant-based | 149 (23.4%) | 1.10 (0.86, 1.41) | 0.446 | 1.02 (0.77, 1.35) | 0.883 |
| Pedicled flap | 26 (4.1%) | 0.81 (0.50, 1.32) | 0.400 | 0.85 (0.50, 1.45) | 0.560 |
| Free flap | 46 (7.2%) | 0.78 (0.64, 0.95) | 0.013 | 0.79 (0.65, 0.96) | 0.018 |
| **Post-operative complications** | **637** |  |  |  |  |
| None | 389 (61.1%) | Reference |  | Reference |  |
| Minor complications | 194 (30.5%) | 0.89 (0.75, 1.06) | 0.184 | 0.93 (0.78, 1.12) | 0.462 |
| Major complications | 54 (8.5%) | 0.66 (0.51, 0.85) | 0.002 | 0.72 (0.54, 0.94) | 0.017 |
| **Age** | **635** | 1.00 (0.99, 1.01) | 0.848 | 1.00 (0.99, 1.01) | 0.839 |
| **BMI** | **615** |  |  |  |  |
| Underweight | 18 (2.9%) | 1.03 (0.66, 1.60) | 0.892 | 0.97 (0.59, 1.58) | 0.894 |
| Normal weight | 224 (36.4%) | Reference |  | Reference |  |
| Overweight | 209 (34.0%) | 0.91 (0.72, 1.15) | 0.407 | 0.89 (0.71, 1.13) | 0.344 |
| Obese | 99 (16.1%) | 0.74 (0.57, 0.95) | 0.018 | 0.75 (0.57, 0.99) | 0.042 |
| Severely obese | 65 (10.6%) | 0.66 (0.50, 0.88) | 0.004 | 0.73 (0.53, 0.99) | 0.042 |
| **Co-morbidities** |  |  |  |  |  |
| **Ischaemic heart disease** | **634** |  |  |  |  |
| No | 618 (97.5%) | Reference |  | Reference |  |
| Yes | 16 (2.5%) | 0.68 (0.48, 0.95) | 0.026 | 0.68 (0.43, 1.08) | 0.100 |
| **Diabetes** | **620** |  |  |  |  |
| No | 573 (92.4%) | Reference |  | Reference |  |
| Yes | 47 (7.6%) | 0.77 (0.60, 0.97) | 0.028 | 0.98 (0.76, 1.26) | 0.869 |
| **Other comorbidity** | **634** |  |  |  |  |
| No | 348 (54.9%) | Reference |  | Reference |  |
| Yes | 286 (45.1%) | 0.88 (0.72, 1.08) | 0.232 | 0.88 (0.70, 1.12) | 0.317 |
| **Smoking status** | **628** |  |  |  |  |
| Non-smoker | 445 (70.9%) | Reference |  | Reference |  |
| Ex-smoker | 93 (14.8%) | 1.11 (0.86, 1.42) | 0.426 | 1.19 (0.93, 1.53) | 0.174 |
| Current smoker | 90 (14.3%) | 0.81 (0.66, 0.98) | 0.034 | 0.86 (0.69, 1.07) | 0.179 |
| **ASA grade** | **634** |  |  |  |  |
| 1 | 231 (36.4%) | Reference |  | Reference |  |
| 2 | 349 (55.1%) | 1.00 (0.85, 1.19) | 0.958 | 1.15 (0.90, 1.46) | 0.265 |
| 3 | 54 (8.5%) | 0.95 (0.72, 1.25) | 0.709 | 1.14 (0.75, 1.73) | 0.529 |
| 4 | 0 (0.0%) | NA | NA | NA |  |
| **Bilateral surgery (vs none)** | **637** | 0.90 (0.69, 1.18) | 0.436 | 0.90 (0.66, 1.22) | 0.484 |

^a^aHR<1 = increased time to adjuvant treatment aHR>1 = shorter time to adjuvant treatment

ASA – American Society of Anaesthesiologists, BMI – body mass index
