# Supplementary material for: The impact of immediate breast reconstruction on the time to delivery of adjuvant therapy: the iBRA-2 study
Source: Br J Cancer. 2019 Mar 29;120(9):883–95. doi: 10.1038/s41416-019-0438-1 (PMC6734656; doi:10.1038/s41416-019-0438-1)
Supplement: Supplementary file 3 — Supplementary table 3 [file 41416_2019_438_MOESM3_ESM.docx]

**Supplementary table 3: Cox univariable and multivariable survival analyses for time to radiotherapy**

|  |  | **Univariable** | | **Multivariable (N=551)** | |
| --- | --- | --- | --- | --- | --- |
|  |  | **Hazard Ratio^a^** |  | **Hazard Ratio^a^** |  |
|  | **N (%)** | **(95% Confidence Intervals)** | **P value** | **(95% Confidence Intervals)** | **P value** |
| **Procedure type** | **6**16 |  |  |  |  |
| Mastectomy only | 411 (66.7%) | Reference |  | Reference |  |
| Implant-based | 134 (21.8%) | 1.02 (0.71, 1.46) | 0.914 | 1.06 (0.76, 1.49) | 0.724 |
| Pedicled flap | 29 (4.7%) | 0.94 (0.59, 1.49) | 0.799 | 0.78 (0.51, 1.21) | 0.274 |
| Free flap | 42 (6.8%) | 1.14 (0.86, 1.50) | 0.354 | 1.06 (0.74, 1.52) | 0.759 |
| **Post-operative complications** | **6**16 |  |  |  |  |
| None | 378 (61.4%) | Reference |  | Reference |  |
| Minor complications | 197 (32.0%) | 0.94 (0.81, 1.10) | 0.467 | 0.87 (0.73, 1.05) | 0.147 |
| Major complications | 41 (6.7%) | 0.77 (0.58, 1.01) | 0.059 | 0.70 (0.53, 0.93) | 0.013 |
| **Age** | **614** | 1.00 (0.99, 1.00) | 0.506 | 1.01 (1.01, 1.02) | <0.001 |
| **BMI** | **577** |  |  |  |  |
| Underweight | 13 (2.3%) | 0.65 (0.33, 1.28) | 0.215 | 0.79 (0.42, 1.46) | 0.447 |
| Normal weight | 202 (35.0%) | Reference |  | Reference |  |
| Overweight | 191 (33.1%) | 0.96 (0.75, 1.22) | 0.717 | 1.00 (0.79, 1.27) | 0.986 |
| Obese | 104 (18.0%) | 0.93 (0.75, 1.16) | 0.527 | 0.86 (0.68, 1.09) | 0.207 |
| Severely obese | 67 (11.6%) | 1.03 (0.80, 1.33) | 0.796 | 1.07 (0.85, 1.33) | 0.571 |
| **Co-morbidities** |  |  |  |  |  |
| **Ischaemic heart disease** | **616** |  |  |  |  |
| No | 581 (94.3%) | Reference |  | Reference |  |
| Yes | 35 (5.7%) | 1.10 (0.76, 1.58) | 0.618 | 1.33 (0.90, 1.97) | 0.155 |
| **Diabetes** | **603** |  |  |  |  |
| No | 538 (89.2%) | Reference |  | Reference |  |
| Yes | 65 (10.8%) | 0.97 (0.77, 1.22) | 0.777 | 1.06 (0.85, 1.31) | 0.619 |
| **Other comorbidity** | **611** |  |  |  |  |
| No | 363 (59.4%) | Reference |  | Reference |  |
| Yes | 248 (40.6%) | 0.90 (0.75, 1.08) | 0.265 | 0.89 (0.70, 1.13) | 0.327 |
| **Smoking status** | **606** |  |  |  |  |
| Non-smoker | 445 (73.4%) | Reference |  | Reference |  |
| Ex-smoker | 92 (15.2%) | 1.02 (0.81, 1.28) | 0.898 | 1.03 (0.83, 1.29) | 0.762 |
| Current smoker | 69 (11.4%) | 0.76 (0.61, 0.96) | 0.022 | 0.73 (0.57, 0.94) | 0.015 |
| **Neoadjuvant chemotherapy** | **614** |  |  |  |  |
| No | 345 (56.2%) | Reference |  | Reference |  |
| Yes | 269 (43.8%) | 2.41 (1.99, 2.93) | <0.001 | 3.09 (2.47, 3.85) | <0.001 |
| **ASA grade** | **613** |  |  |  |  |
| 1 | 174 (28.4%) | Reference |  | Reference |  |
| 2 | 369 (60.2%) | 0.99 (0.83, 1.19) | 0.955 | 0.89 (0.69, 1.16) | 0.387 |
| 3 | 68 (11.1%) | 1.06 (0.84, 1.33) | 0.645 | 1.12 (0.81, 1.55) | 0.481 |
| 4 | 2 (0.3%) | 1.44 (1.15, 1.80) | 0.001 | 0.74 (0.27, 2.01) | 0.558 |
| **Bilateral surgery (vs none)** | **616** | 1.20 (0.97, 1.49) | 0.094 | 1.09 (0.88, 1.34) | 0.438 |

^a^aHR<1 = increased time to adjuvant treatment aHR>1 = shorter time to adjuvant treatment

ASA – American Society of Anaesthesiologists, BMI – body mass index
