# Supplementary material for: The impact of immediate breast reconstruction on the time to delivery of adjuvant therapy: the iBRA-2 study
Source: Br J Cancer. 2019 Mar 29;120(9):883–95. doi: 10.1038/s41416-019-0438-1 (PMC6734656; doi:10.1038/s41416-019-0438-1)
Supplement: Supplementary file 4 — Supplementary table 4 [file 41416_2019_438_MOESM4_ESM.docx]

**Supplementary table 4: Time to adjuvant therapy by whether the patient had no, minor, or major complications**

|  | **No complications (n=1564)** | **Minor complications (n=755)** | **Major complications (n=221)** | **P value** |
| --- | --- | --- | --- | --- |
| **Patient accepts adjuvant treatment (either chemotherapy or radiotherapy or both)** | 736 (47.1) | 398 (52.7) | 101 (45.7) | 0.025 |
| **Time from last oncological procedure to first adjuvant treatment (days) median (IQR) (n=1131)** | 50 (39-63) | 56 (42.5-69) | 57 (46-73) | <0.001 |
| **Chemotherapy as 1st adjuvant treatment** | 382 (55.8) | 191 (53.1) | 54 (62.8) | 0.254 |
| **Time from last oncological procedure to chemotherapy (days) median (IQR) (n=637)** | 45 (35-59) | 49 (39-60) | 51 (45-68) | <0.001 |
| **Planned chemotherapy delayed by >90 days** | 14 (3.6) | 10 (5.2) | 7 (13.0) | 0.011 |
| **Radiotherapy as 1st adjuvant treatment** | 303 (44.2) | 169 (46.9) | 32 (37.2) | 0.254 |
| **Time from last oncological procedure to radiotherapy (days) median (IQR) (n=616)** | 62 (47-82) | 66 (55-86) | 72 (54-130) | 0.026 |
| **Planned radiotherapy delayed by >56 days (8 weeks)** | 222 (58.7) | 138 (70.1) | 29 (70.7) | 0.016 |

IQR – interquartile range; MDT – multidisciplinary team
